# Bis(μ-4,6-dimethyl­pyrimidine-2-thiol­ato)-κ^3^ *N*,*S*:*S*;κ^3^ *S*:*N*,*S*-bis­[(triphenyl­phosphane-κ*P*)silver(I)]

**DOI:** 10.1107/S1600536812048210

**Published:** 2012-11-30

**Authors:** Yupa Wattanakanjana, Chaveng Pakawatchai, Sukanya Kowittheeraphong, Ruthairat Nimthong

**Affiliations:** a Department of Chemistry, Faculty of Science, Prince of Songkla University, Hat Yai, Songkhla 90112, Thailand

## Abstract

The dinuclear title complex, [Ag_2_(C_6_H_7_N_2_S)_2_(C_18_H_15_P)_2_], comprises two inversion-related [Ag(C_6_H_7_N_2_S)(C_18_H_15_P)] units. The pyrimidine­thiol­ate anion acts both as a bridging and a chelating ligand. The Ag^I^ ions are linked *via* two *μ*_2_-S donor atoms, which generate a strictly planar Ag_2_S_2_ core with an Ag⋯Ag separation of 2.9569 (4) Å. The Ag^I^ ion presents a distorted tetra­hedral coordination geometry. In the crystal, weak C—H⋯N and C—H⋯S hydrogen bonds link the complex mol­ecules into a two-dimensional network parallel to (010).

## Related literature

For the structures of metal(I) coordination compounds and their potential applications, see: Aslanidis *et al.* (1997 ▶); McFarlane *et al.* (1998 ▶); Nawaz *et al.* (2011 ▶); Hameau *et al.* (2012 ▶); Nimthong *et al.* (2012 ▶); Pakawatchai *et al.* (2012 ▶). For relevant examples of discrete complexes, see: Cox *et al.* (2000 ▶); Lobana *et al.* (2008 ▶); Isab *et al.* (2010 ▶).
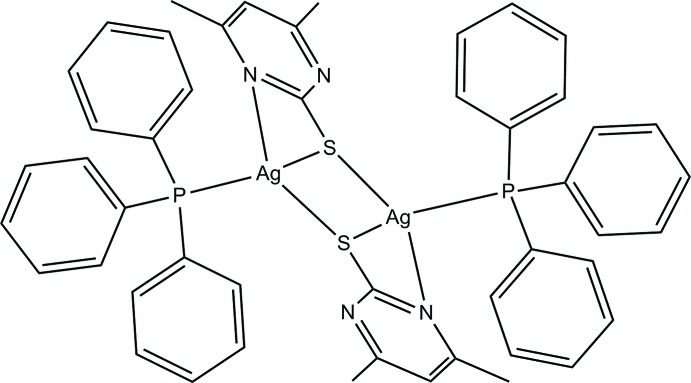


## Experimental

### 

#### Crystal data

[Ag_2_(C_6_H_7_N_2_S)_2_(C_18_H_15_P)_2_]
*M* *_r_* = 1018.67
Monoclinic, 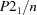

*a* = 11.7050 (5) Å
*b* = 15.3084 (7) Å
*c* = 12.5331 (6) Å
β = 97.483 (1)°
*V* = 2226.62 (18) Å^3^
*Z* = 2
Mo *K*α radiation
μ = 1.08 mm^−1^
*T* = 293 K
0.29 × 0.18 × 0.09 mm

#### Data collection

Bruker SMART CCD diffractometer
Absorption correction: multi-scan (*SADABS*; Bruker, 2003 ▶) *T*_min_ = 0.793, *T*_max_ = 0.909
26686 measured reflections
5568 independent reflections
4798 reflections with *I* > 2σ(*I*)
*R*_int_ = 0.023

#### Refinement

*R*[*F* ^2^ > 2σ(*F* ^2^)] = 0.032
*wR*(*F* ^2^) = 0.088
*S* = 1.04
5568 reflections
266 parameters
H atoms treated by a mixture of independent and constrained refinement
Δρ_max_ = 0.54 e Å^−3^
Δρ_min_ = −0.29 e Å^−3^

### 

Data collection: *SMART* (Bruker, 1998 ▶); cell refinement: *SAINT* (Bruker, 2003 ▶); data reduction: *SAINT*; program(s) used to solve structure: *SHELXS97* (Sheldrick, 2008 ▶); program(s) used to refine structure: *SHELXL97* (Sheldrick, 2008 ▶); molecular graphics: *Mercury* (Macrae *et al.*, 2008 ▶); software used to prepare material for publication: *SHELXTL* (Sheldrick, 2008 ▶) and *publCIF* (Westrip, 2010 ▶).

## Supplementary Material

Crystal structure: contains datablock(s) I, global. DOI: 10.1107/S1600536812048210/bh2464sup1.cif

Structure factors: contains datablock(s) I. DOI: 10.1107/S1600536812048210/bh2464Isup2.hkl

Additional supplementary materials:  crystallographic information; 3D view; checkCIF report

## Figures and Tables

**Table 1:** Hydrogen-bond geometry (Å, °)

*D*—H⋯*A*	*D*—H	H⋯*A*	*D*⋯*A*	*D*—H⋯*A*
C14—H14⋯S1^i^	0.93	2.94	3.801 (3)	154
C14—H14⋯N2^ii^	0.93	2.69	3.471 (4)	143
C35—H35⋯N2^iii^	0.93	2.93	3.756 (4)	151

Symmetry codes: (i) 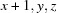; (ii) 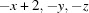; (iii) 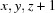.

**Fig. 1.**
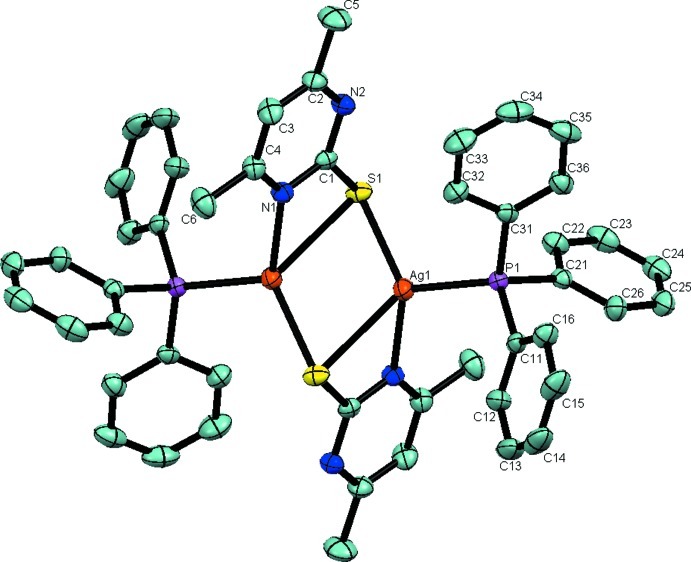
The molecular structure with displacement ellipsoids drawn at the 30% probability level.

**Fig. 2.**
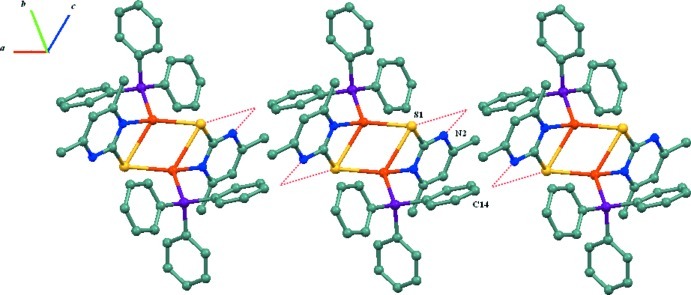
Part of the crystal structure with C—H···N and C—H···S hydrogen bonds interactions showed as dashed lines.

**Fig. 3.**
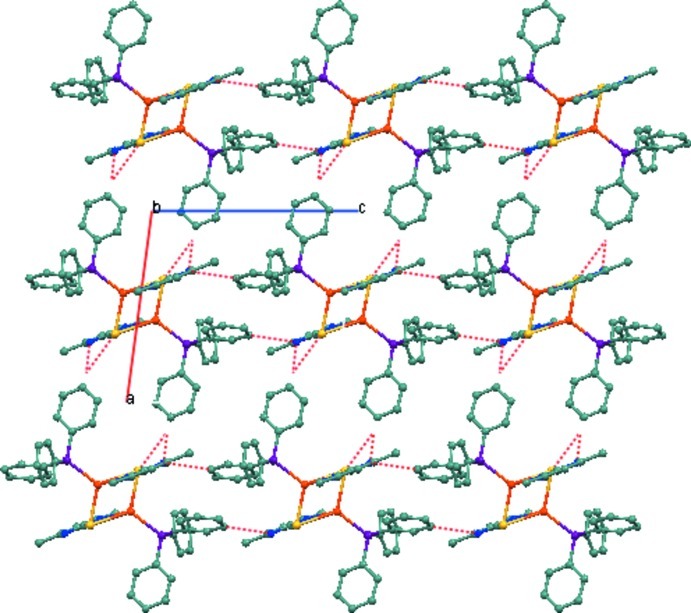
The packing diagram viewed down the b axis. The dashed lines represent intermolecular C—H···N and C—H···S interactions.

## supplementary crystallographic information

### Comment

In recent years, a large number of structural reports on metal(I) complexes containing heterocyclic thiones as ligands or mixed-ligands with triphenylphosphane have been studied (Aslanidis *et al.*, 1997; McFarlane *et al.*, 1998; Pakawatchai *et al.*, 2012; Nimthong *et al.*, 2012) because of not only their potential applications due to their antimicrobial activities (Nawaz *et al.*, 2011), but also strongly luminescent properties (Hameau *et al.*, 2012).

The structure of the title dinuclear mixed-ligand complex displays the distorted tetrahedral coordination of each Ag^I^ center, which exhibits a planar Ag_2_S_2_ moiety in which each of the doubly S-bridged Ag^I^ centers is surrounded by the one P atom of phosphane ligand and one N atom of the dmpymtH ligands (Fig. 1). The Ag—Ag distance of 2.9569 (4) Å in the four-membered Ag_2_S_2_ ring is shorter than in [Ag_2_*X*_2_(l-S-pySH)_2_(PPh_3_)_2_] (*X* = Cl and Br), 3.8425 (8) and 3.8211 (4) Å, respectively (Lobana *et al.*, 2008) and also shorter than the sum of the covalent radii of two Ag^I^ centers (3.44 Å). Focusing on the comparison of bond distances and bond angles around the Ag^I^ ion, the Ag—S bond lengths [2.5492 (6)–2.7897 (6) Å] are in good agreement with values reported for other silver(I) complexes with heterocyclic thione ligands, such as 2.5548 (9) Å for [Ag(PPh_3_)(pymtH)Br]_2_ (Cox *et al.*, 2000) and 2.537 (2) Å for [Ag(Ph_3_P)(Diaz)_2_]_2_(NO_3_)_2_ (Nawaz *et al.*, 2011). The Ag1—P1 bond length of 2.4088 (6) Å is similar to that found in [Ag(PPh_3_)(thiourea)(NO_3_)]_2_.[Ag(PPh_3_)(thiourea)]_2_(NO_3_)_2_ [2.4029 (10)–2.4157 (10) Å] (Isab *et al.*, 2010). The two S1–Ag1–P1 angles of 116.81 (2) and 123.56 (2)° are larger than the normal tetrahedral value of 109.5°. In the crystal, the intermolecular interactions C14(*sp^2^*)—H14···N2 [H14···N2 = 2.686 (4) Å, C14(*sp^2^*)···N2 = 3.471 (4) Å and C14(*sp^2^*)—H14···N2 = 142.52 (8)°] and C14(*sp^2^*)—H14···S1 [H14···S1 = 2.942 (3) Å, C14(*sp^2^*)···S1 = 3.801 (3) Å and C14(*sp^2^*)—H14···S1 = 154.18 (9)°] form chains (Fig. 2). Moreover, secondary interactions C35(*sp^2^*)—H35···N2 [H35···N2 = 2.928 (4) Å, C35(*sp^2^*)···N2 = 3.756 (4) Å and C35(*sp^2^*)—H35···N2 = 150.48 (7)°] are also observed, which form the two-dimensional layer network (Fig. 3).

### Experimental

Triphenylphosphane (0.31 g, 1.18 mmol) was dissolved in 30 cm^3^ of ethanol at 335 K. Silver acetate (0.10 g, 0.60 mmol) was added and the mixture was stirred for 3 h. 4,6-Dimethylpyrimidine-2(1*H*)-thione (0.18 g, 0.46 mmol) was added and the new reaction mixture was refluxed for 2 h where upon the precipitate gradually disappeared. The resulting clear solution was filtered off and left to evaporate at room temperature. The crystalline solid, which was deposited upon standing for several days, was filtered off and dried under reduced pressure.

### Refinement

The H atoms bonded to C atoms were constrained to ride on their parent atoms with C—H bond lengths of 0.93 Å [aryl CH, *U*_iso_(H) = 1.2*U*_eq_(C)] and 0.96 Å [methyl CH_3_, *U*_iso_(H) = 1.5*U*_eq_(C)] except for H3, which was located in a difference map and refined isotropically.

### Crystal data

**Table:**

[Ag_2_(C_6_H_7_N_2_S)_2_(C_18_H_15_P)_2_]	*F*(000) = 1032
*M**_r_* = 1018.67	*D*_x_ = 1.519 Mg m^−^^3^
Monoclinic, *P*2_1_/*n*	Mo *K*α radiation, λ = 0.71073 Å
Hall symbol: -P 2yn	Cell parameters from 8845 reflections
*a* = 11.7050 (5) Å	θ = 2.2–28.3°
*b* = 15.3084 (7) Å	µ = 1.08 mm^−^^1^
*c* = 12.5331 (6) Å	*T* = 293 K
β = 97.483 (1)°	Block, yellow
*V* = 2226.62 (18) Å^3^	0.29 × 0.18 × 0.09 mm
*Z* = 2	

### Data collection

**Table:**

Bruker SMART CCD diffractometer	5568 independent reflections
Radiation source: fine-focus sealed tube	4798 reflections with *I* > 2σ(*I*)
Graphite monochromator	*R*_int_ = 0.023
φ and ω scans	θ_max_ = 28.3°, θ_min_ = 2.1°
Absorption correction: multi-scan (*SADABS*; Bruker, 2003)	*h* = −15→15
*T*_min_ = 0.793, *T*_max_ = 0.909	*k* = −20→20
26686 measured reflections	*l* = −16→16

### Refinement

**Table:**

Refinement on *F*^2^	Primary atom site location: structure-invariant direct methods
Least-squares matrix: full	Secondary atom site location: difference Fourier map
*R*[*F*^2^ > 2σ(*F*^2^)] = 0.032	Hydrogen site location: inferred from neighbouring sites
*wR*(*F*^2^) = 0.088	H atoms treated by a mixture of independent and constrained refinement
*S* = 1.04	*w* = 1/[σ^2^(*F*_o_^2^) + (0.0481*P*)^2^ + 0.8969*P*] where *P* = (*F*_o_^2^ + 2*F*_c_^2^)/3
5568 reflections	(Δ/σ)_max_ = 0.003
266 parameters	Δρ_max_ = 0.54 e Å^−^^3^
0 restraints	Δρ_min_ = −0.29 e Å^−^^3^
0 constraints	

### Fractional atomic coordinates and isotropic or equivalent isotropic displacement parameters (Å^2^)

**Table:**

	*x*	*y*	*z*	*U*_iso_*/*U*_eq_	
C1	0.64391 (17)	0.09032 (14)	−0.12789 (17)	0.0376 (4)	
C2	0.6887 (2)	0.20334 (17)	−0.2336 (2)	0.0512 (6)	
C3	0.6524 (3)	0.26179 (17)	−0.1622 (2)	0.0578 (7)	
C4	0.6137 (2)	0.23054 (15)	−0.07073 (19)	0.0457 (5)	
C5	0.7338 (4)	0.2323 (2)	−0.3350 (3)	0.0798 (10)	
H5A	0.7548	0.1820	−0.3739	0.120*	
H5B	0.8003	0.2687	−0.3168	0.120*	
H5C	0.6752	0.2647	−0.3790	0.120*	
C6	0.5799 (3)	0.28888 (19)	0.0155 (2)	0.0673 (8)	
H6A	0.5556	0.2540	0.0721	0.101*	
H6B	0.5177	0.3261	−0.0141	0.101*	
H6C	0.6446	0.3241	0.0440	0.101*	
C21	0.69001 (19)	0.17384 (14)	0.30847 (17)	0.0390 (4)	
C22	0.5829 (2)	0.21344 (18)	0.3033 (2)	0.0525 (6)	
H22	0.5174	0.1845	0.2712	0.063*	
C23	0.5735 (3)	0.2965 (2)	0.3463 (2)	0.0662 (8)	
H23	0.5015	0.3229	0.3427	0.079*	
C24	0.6690 (3)	0.33977 (17)	0.3938 (2)	0.0654 (8)	
H24	0.6618	0.3951	0.4230	0.079*	
C25	0.7747 (3)	0.30172 (18)	0.3984 (2)	0.0620 (7)	
H25	0.8396	0.3314	0.4304	0.074*	
C26	0.7861 (2)	0.21889 (17)	0.3557 (2)	0.0533 (6)	
H26	0.8587	0.1936	0.3588	0.064*	
C31	0.66300 (17)	−0.00663 (15)	0.36218 (17)	0.0384 (4)	
C32	0.6224 (2)	−0.08985 (19)	0.3347 (2)	0.0581 (6)	
H32	0.6112	−0.1070	0.2630	0.070*	
C33	0.5986 (3)	−0.1472 (2)	0.4139 (3)	0.0799 (10)	
H33	0.5735	−0.2035	0.3955	0.096*	
C34	0.6118 (3)	−0.1213 (3)	0.5202 (3)	0.0781 (10)	
H34	0.5955	−0.1602	0.5732	0.094*	
C35	0.6487 (3)	−0.0385 (2)	0.5481 (2)	0.0698 (9)	
H35	0.6559	−0.0207	0.6196	0.084*	
C36	0.6751 (2)	0.0184 (2)	0.4697 (2)	0.0530 (6)	
H36	0.7013	0.0743	0.4890	0.064*	
N1	0.60828 (16)	0.14406 (12)	−0.05384 (15)	0.0397 (4)	
N2	0.68507 (17)	0.11646 (13)	−0.21745 (15)	0.0452 (4)	
H3	0.665 (3)	0.318 (2)	−0.173 (2)	0.064 (8)*	
C11	0.84851 (19)	0.04687 (14)	0.2463 (2)	0.0406 (5)	
C12	0.8906 (3)	0.0742 (2)	0.1534 (2)	0.0591 (7)	
H12	0.8425	0.1018	0.0987	0.071*	
C13	1.0069 (3)	0.0597 (2)	0.1432 (3)	0.0782 (11)	
H13	1.0360	0.0783	0.0815	0.094*	
C14	1.0778 (3)	0.0188 (2)	0.2223 (4)	0.0801 (11)	
H14	1.1547	0.0093	0.2143	0.096*	
C15	1.0368 (2)	−0.0081 (2)	0.3123 (3)	0.0726 (9)	
H15	1.0858	−0.0362	0.3658	0.087*	
C16	0.9221 (2)	0.00566 (18)	0.3264 (2)	0.0531 (6)	
H16	0.8950	−0.0127	0.3892	0.064*	
Ag1	0.570898 (16)	0.048363 (12)	0.086830 (13)	0.04660 (8)	
P1	0.69618 (5)	0.06407 (4)	0.25410 (4)	0.03501 (12)	
S1	0.35819 (5)	0.02130 (4)	0.09946 (5)	0.04512 (14)	

### Atomic displacement parameters (Å^2^)

**Table:**

	*U*^11^	*U*^22^	*U*^33^	*U*^12^	*U*^13^	*U*^23^
C1	0.0319 (9)	0.0395 (11)	0.0411 (11)	0.0003 (8)	0.0034 (8)	0.0072 (9)
C2	0.0592 (15)	0.0507 (14)	0.0446 (12)	−0.0067 (11)	0.0096 (11)	0.0118 (11)
C3	0.083 (2)	0.0362 (13)	0.0531 (15)	−0.0049 (12)	0.0057 (13)	0.0099 (11)
C4	0.0538 (13)	0.0383 (11)	0.0436 (12)	0.0023 (10)	0.0012 (10)	0.0032 (9)
C5	0.113 (3)	0.070 (2)	0.0621 (18)	−0.0094 (19)	0.0349 (18)	0.0209 (16)
C6	0.097 (2)	0.0480 (15)	0.0575 (16)	0.0074 (15)	0.0102 (16)	−0.0049 (12)
C21	0.0473 (11)	0.0363 (10)	0.0345 (10)	0.0001 (9)	0.0090 (9)	0.0024 (8)
C22	0.0541 (14)	0.0543 (14)	0.0499 (13)	0.0089 (11)	0.0103 (11)	0.0040 (11)
C23	0.082 (2)	0.0571 (16)	0.0636 (17)	0.0293 (16)	0.0268 (16)	0.0113 (14)
C24	0.108 (2)	0.0344 (12)	0.0598 (16)	0.0041 (14)	0.0351 (17)	0.0037 (11)
C25	0.084 (2)	0.0435 (14)	0.0615 (16)	−0.0152 (14)	0.0203 (15)	−0.0097 (12)
C26	0.0585 (15)	0.0439 (13)	0.0584 (15)	−0.0052 (11)	0.0108 (12)	−0.0087 (11)
C31	0.0330 (10)	0.0453 (12)	0.0378 (10)	0.0022 (9)	0.0075 (8)	0.0037 (9)
C32	0.0635 (16)	0.0516 (15)	0.0603 (16)	−0.0116 (13)	0.0116 (13)	0.0003 (12)
C33	0.083 (2)	0.0578 (18)	0.100 (3)	−0.0168 (16)	0.019 (2)	0.0209 (18)
C34	0.0669 (19)	0.090 (2)	0.081 (2)	0.0028 (17)	0.0261 (17)	0.041 (2)
C35	0.0621 (17)	0.105 (3)	0.0443 (14)	0.0128 (17)	0.0149 (13)	0.0211 (15)
C36	0.0534 (14)	0.0641 (16)	0.0412 (12)	0.0022 (12)	0.0047 (11)	0.0015 (11)
N1	0.0436 (9)	0.0373 (9)	0.0383 (9)	0.0003 (8)	0.0061 (7)	0.0036 (7)
N2	0.0486 (10)	0.0472 (11)	0.0415 (10)	−0.0010 (9)	0.0118 (8)	0.0043 (8)
C11	0.0376 (11)	0.0377 (11)	0.0481 (12)	−0.0057 (8)	0.0120 (9)	−0.0107 (9)
C12	0.0602 (16)	0.0659 (16)	0.0554 (15)	−0.0148 (13)	0.0236 (13)	−0.0094 (13)
C13	0.071 (2)	0.087 (2)	0.087 (2)	−0.0274 (18)	0.047 (2)	−0.0282 (19)
C14	0.0470 (15)	0.071 (2)	0.129 (3)	−0.0104 (15)	0.036 (2)	−0.039 (2)
C15	0.0417 (14)	0.0596 (18)	0.116 (3)	0.0051 (13)	0.0075 (16)	−0.0152 (18)
C16	0.0423 (12)	0.0517 (14)	0.0653 (16)	0.0035 (11)	0.0071 (11)	−0.0039 (12)
Ag1	0.05306 (12)	0.05106 (12)	0.03463 (10)	−0.01141 (8)	0.00173 (8)	0.00047 (7)
P1	0.0351 (3)	0.0382 (3)	0.0317 (3)	−0.0029 (2)	0.0043 (2)	−0.0018 (2)
S1	0.0448 (3)	0.0354 (3)	0.0576 (3)	0.0031 (2)	0.0160 (3)	0.0063 (2)

### Geometric parameters (Å, º)

**Table:**

C1—N2	1.339 (3)	C31—P1	1.815 (2)
C1—N1	1.346 (3)	C32—C33	1.380 (4)
C1—S1^i^	1.746 (2)	C32—H32	0.9300
C2—N2	1.347 (3)	C33—C34	1.379 (5)
C2—C3	1.372 (4)	C33—H33	0.9300
C2—C5	1.505 (3)	C34—C35	1.370 (5)
C3—C4	1.373 (3)	C34—H34	0.9300
C3—H3	0.89 (3)	C35—C36	1.377 (4)
C4—N1	1.343 (3)	C35—H35	0.9300
C4—C6	1.495 (4)	C36—H36	0.9300
C5—H5A	0.9600	N1—Ag1	2.3763 (18)
C5—H5B	0.9600	C11—C12	1.386 (3)
C5—H5C	0.9600	C11—C16	1.387 (4)
C6—H6A	0.9600	C11—P1	1.818 (2)
C6—H6B	0.9600	C12—C13	1.401 (4)
C6—H6C	0.9600	C12—H12	0.9300
C21—C26	1.385 (3)	C13—C14	1.360 (6)
C21—C22	1.387 (3)	C13—H13	0.9300
C21—P1	1.818 (2)	C14—C15	1.346 (5)
C22—C23	1.391 (4)	C14—H14	0.9300
C22—H22	0.9300	C15—C16	1.393 (4)
C23—C24	1.367 (5)	C15—H15	0.9300
C23—H23	0.9300	C16—H16	0.9300
C24—C25	1.362 (5)	Ag1—P1	2.4088 (6)
C24—H24	0.9300	Ag1—S1	2.5492 (6)
C25—C26	1.390 (4)	Ag1—S1^i^	2.7897 (6)
C25—H25	0.9300	Ag1—Ag1^i^	2.9569 (4)
C26—H26	0.9300	S1—C1^i^	1.746 (2)
C31—C32	1.387 (4)	S1—Ag1^i^	2.7897 (6)
C31—C36	1.390 (3)		
			
N2—C1—N1	125.0 (2)	C35—C34—C33	120.3 (3)
N2—C1—S1^i^	118.66 (17)	C35—C34—H34	119.9
N1—C1—S1^i^	116.31 (15)	C33—C34—H34	119.9
N2—C2—C3	121.8 (2)	C34—C35—C36	119.7 (3)
N2—C2—C5	116.1 (3)	C34—C35—H35	120.2
C3—C2—C5	122.1 (3)	C36—C35—H35	120.2
C2—C3—C4	118.8 (2)	C35—C36—C31	120.9 (3)
C2—C3—H3	117.6 (19)	C35—C36—H36	119.6
C4—C3—H3	123 (2)	C31—C36—H36	119.6
N1—C4—C3	120.1 (2)	C4—N1—C1	117.93 (19)
N1—C4—C6	116.9 (2)	C4—N1—Ag1	137.84 (16)
C3—C4—C6	122.9 (2)	C1—N1—Ag1	103.78 (13)
C2—C5—H5A	109.5	C1—N2—C2	116.3 (2)
C2—C5—H5B	109.5	C12—C11—C16	119.3 (2)
H5A—C5—H5B	109.5	C12—C11—P1	117.4 (2)
C2—C5—H5C	109.5	C16—C11—P1	123.23 (18)
H5A—C5—H5C	109.5	C11—C12—C13	119.0 (3)
H5B—C5—H5C	109.5	C11—C12—H12	120.5
C4—C6—H6A	109.5	C13—C12—H12	120.5
C4—C6—H6B	109.5	C14—C13—C12	120.9 (3)
H6A—C6—H6B	109.5	C14—C13—H13	119.5
C4—C6—H6C	109.5	C12—C13—H13	119.5
H6A—C6—H6C	109.5	C15—C14—C13	120.1 (3)
H6B—C6—H6C	109.5	C15—C14—H14	120.0
C26—C21—C22	118.8 (2)	C13—C14—H14	120.0
C26—C21—P1	123.42 (18)	C14—C15—C16	121.0 (3)
C22—C21—P1	117.80 (19)	C14—C15—H15	119.5
C21—C22—C23	119.9 (3)	C16—C15—H15	119.5
C21—C22—H22	120.1	C11—C16—C15	119.7 (3)
C23—C22—H22	120.1	C11—C16—H16	120.2
C24—C23—C22	120.7 (3)	C15—C16—H16	120.2
C24—C23—H23	119.7	N1—Ag1—P1	115.73 (5)
C22—C23—H23	119.7	N1—Ag1—S1	114.96 (5)
C25—C24—C23	119.8 (3)	P1—Ag1—S1	116.81 (2)
C25—C24—H24	120.1	N1—Ag1—S1^i^	60.73 (5)
C23—C24—H24	120.1	P1—Ag1—S1^i^	123.56 (2)
C24—C25—C26	120.4 (3)	S1—Ag1—S1^i^	112.912 (15)
C24—C25—H25	119.8	N1—Ag1—Ag1^i^	84.41 (5)
C26—C25—H25	119.8	P1—Ag1—Ag1^i^	155.550 (17)
C21—C26—C25	120.4 (3)	S1—Ag1—Ag1^i^	60.342 (16)
C21—C26—H26	119.8	S1^i^—Ag1—Ag1^i^	52.570 (14)
C25—C26—H26	119.8	C31—P1—C11	105.11 (10)
C32—C31—C36	118.8 (2)	C31—P1—C21	104.39 (10)
C32—C31—P1	117.53 (18)	C11—P1—C21	103.96 (10)
C36—C31—P1	123.68 (19)	C31—P1—Ag1	115.14 (7)
C33—C32—C31	120.0 (3)	C11—P1—Ag1	115.39 (8)
C33—C32—H32	120.0	C21—P1—Ag1	111.66 (7)
C31—C32—H32	120.0	C1^i^—S1—Ag1	102.21 (7)
C34—C33—C32	120.3 (3)	C1^i^—S1—Ag1^i^	79.07 (7)
C34—C33—H33	119.9	Ag1—S1—Ag1^i^	67.088 (15)
C32—C33—H33	119.9		

Symmetry code: (i) −*x*+1, −*y*, −*z*.

### Hydrogen-bond geometry (Å, º)

**Table:**

*D*—H···*A*	*D*—H	H···*A*	*D*···*A*	*D*—H···*A*
C14—H14···S1^ii^	0.93	2.94	3.801 (3)	154
C14—H14···N2^iii^	0.93	2.69	3.471 (4)	143
C35—H35···N2^iv^	0.93	2.93	3.756 (4)	151

Symmetry codes: (ii) *x*+1, *y*, *z*; (iii) −*x*+2, −*y*, −*z*; (iv) *x*, *y*, *z*+1.
